# Incorporating Sales Value into Weight-Based Veterinary Antimicrobial Use Surveillance: An Analysis of Veterinary Antimicrobial Sales Data in Japan, 2011–2024

**DOI:** 10.3390/antibiotics15090908

**Published:** 2026-09-15

**Authors:** Yuta Hosoi, Mari Matsuda, Michiko Kawanishi, Saki Harada, Shunya Okamura, Tetsuo Asai, Hideto Sekiguchi

**Affiliations:** 1 Veterinary AMR Center, National Veterinary Assay Laboratory, Ministry of Agriculture, Forestry and Fisheries, Tsukuba 305-8535, Japan; 2 The United Graduate School of Veterinary Sciences, Gifu University, 1-1 Yanagito, Gifu 501-1193, Japan

**Keywords:** veterinary antimicrobial agents, AMR, AMU, JVARM, elasticity, One Health, surveillance

## Abstract

**Background/Objectives:** Veterinary antimicrobial surveillance has been developed mainly around weight-based indicators, whereas the monetary dimension of antimicrobial sales has been less commonly reported. The Japanese Veterinary Antimicrobial Resistance Monitoring System (JVARM) records both sales volume and sales value, enabling the quantity and monetary dimensions of antimicrobial sales to be examined together. The objective of this study was to assess whether examining sales value alongside sales volume could provide additional surveillance information on their temporal trends and distributions across antimicrobial classes and animal species. **Methods:** We extracted annual veterinary antimicrobial sales data for 2011–2024 from publicly available JVARM reports. We summarized sales volume and sales value overall, by antimicrobial class, by animal category, and by antimicrobial class within each animal category. We examined their joint patterns using two-dimensional volume–value positioning and standardized annual class-specific trajectories and estimated class-specific elasticity and marginal effects using an implied unit price calculated as sales value divided by sales volume. **Results:** Total sales volume increased from 794 tonnes in 2011 to a peak of 859 tonnes in 2017 and then declined to 745 tonnes in 2024, whereas total sales value increased overall from JPY 19.1 billion in 2011 to JPY 28.7 billion in 2024. In 2024, tetracyclines remained the dominant class by volume at 292 tonnes, whereas cephalosporins, macrolides, and fluoroquinolones were the leading classes by sales value at JPY 4.73, 4.55, and 4.54 billion, respectively. In 2024, pigs ranked first among animal sectors in both sales volume and sales value, at 363 tonnes and JPY 10.28 billion, respectively. Dogs and cats accounted for only 8.6 tonnes but ranked second in sales value at JPY 7.32 billion. The volume–value plots illustrated contrasting positions of antimicrobial classes: tetracyclines, macrolides, and penicillins were located in the high-volume/high-value quadrant; sulfonamides in the high-volume/low-value quadrant; and cephalosporins and fluoroquinolones in the low-volume/high-value quadrant. Standardized trajectories showed concordant movement for some classes but marked divergence for others, most notably tetracyclines, for which sales volume declined while sales value increased. Cephalosporins and fluoroquinolones showed relatively weak proportional responsiveness, with elasticity estimates of −0.70 and −1.00 and marginal effect estimates of −0.03 and −0.05 kg per JPY/g. In contrast, sulfonamides and penicillins showed stronger proportional responsiveness, with elasticity estimates of −1.65 and −1.67, respectively. **Conclusions:** The joint analysis of sales volume and sales value revealed differences between the two measures in temporal trajectories and the relative prominence of antimicrobial classes and animal sectors. Veterinary antimicrobial use surveillance may therefore benefit from analyzing sales value alongside established sales volume measures, as this approach provides complementary market context.

## 1. Introduction

Antimicrobial resistance (AMR) is widely recognised as one of the major global health challenges of our time. In 2015, the World Health Assembly endorsed the World Health Organization (WHO) Global Action Plan on Antimicrobial Resistance, which identified surveillance and research, as well as the optimisation of antimicrobial use in both human and animal health, among its core strategic objectives [1]. A monitoring and evaluation framework subsequently co-developed by the WHO, the Food and Agriculture Organization of the United Nations (FAO), and the World Organisation for Animal Health (WOAH, formerly OIE) further included antimicrobial consumption among the recommended indicators for monitoring progress under the Global Action Plan [2]. In the veterinary sector, these international initiatives have stimulated the development of national and supranational systems for monitoring antimicrobial use (AMU) in animals. More recently, the Quadripartite organizations—FAO, UNEP, WHO and WOAH—updated the Global Action Plan on Antimicrobial Resistance for 2026–2036, which was adopted by the World Health Assembly in 2026. The updated plan states that “trends in AMR and antimicrobial use should be consistently tracked and complemented by behavioural and socioeconomic data to enhance understanding of AMR drivers,” thereby more explicitly recognising the role of behavioural and socioeconomic information as contextual data for interpreting AMR and AMU surveillance trends [3].

To date, veterinary AMU surveillance has been built primarily on indicators based on the amount of antimicrobial active ingredient. At the international level, WOAH reports antimicrobial quantities in animals and normalises them by estimated animal biomass, expressing use as milligrams of active ingredient per kilogram of animal biomass (mg/kg biomass) in its annual AMU reports [4]. Similar biomass-adjusted approaches have been adopted in many national and regional surveillance systems. In Europe, the former European Surveillance of Veterinary Antimicrobial Consumption (ESVAC) framework used the population correction unit (PCU) as a biomass-based denominator [5], whereas the current European Sales and Use of Antimicrobials for Veterinary Medicine (ESUAvet) framework uses a different animal-biomass-based denominator for reporting [6]. Other jurisdictions have developed related approaches: Canada reports veterinary antimicrobial sales both in kilograms and in biomass-adjusted indicators, including PCU- and biomass-based measures [7]; Thailand has reported antimicrobial consumption in mg/PCU_Thailand_ [8]; and the United States Food and Drug Administration (FDA) uses target animal biomass (TAB) to generate biomass-adjusted sales indicators for major food-producing species [9].

Beyond simple mass-based metrics, dose-based indicators have been developed to better approximate animal exposure to antimicrobials. The European Medicines Agency (EMA) established standardised technical units such as the defined daily dose for animals (DDDvet) and the defined course dose for animals (DCDvet) to support harmonised reporting across species in Europe [10]. At the national level, terminology and implementation vary. The Netherlands applies a nationally defined dose-based metric, the Defined Daily Dose Animal (DDDA), for benchmarking antimicrobial use at farm and sector levels [11]. In France, antimicrobial exposure is commonly expressed using the Animal Level of Exposure to Antimicrobials (ALEA), in which the treated body weight is estimated by taking into account the dosage and duration of administration of each medicine and then divided by the animal biomass of the animal population potentially exposed to antibiotics over the year [12]. Together, these developments show that veterinary AMU surveillance has evolved by refining denominators and dose concepts in order to approximate exposure more closely; however, the underlying basis of these indicators remains the quantity of antimicrobial substances used or sold.

In Japan, antimicrobial resistance represents a substantial public health burden. Bloodstream infections caused by methicillin-resistant *Staphylococcus aureus* and fluoroquinolone-resistant *Escherichia coli* were estimated to be associated with more than 8000 human deaths in 2017 [13]. Japan’s National Action Plan on Antimicrobial Resistance emphasizes an integrated One Health approach involving human and veterinary medicine, livestock and aquaculture, food safety, and the environment [14]. Within this framework, veterinary AMR and AMU surveillance is conducted through the Japanese Veterinary Antimicrobial Resistance Monitoring System (JVARM), which has operated since 1999 as a national monitoring program covering antimicrobial sales and resistance in bacteria from both healthy and diseased animals [15]. Within JVARM, annual sales data are collected from marketing authorization holders and include the name of the antimicrobial, sales volume expressed as the weight of active ingredients, sales value in Japanese yen (JPY), route of administration, and estimated species allocation. Published national summaries present antimicrobial sales mainly in terms of active ingredient weight [16]. More recently, our group applied biomass-adjusted indicators to Japanese data, showing that interpretation can differ substantially when antimicrobial sales are normalised by animal biomass rather than assessed solely by tonnage [17]. Thus, as in many other countries and international reporting systems, veterinary AMU in Japan has been monitored primarily through weight-based metrics.

While mass-based indicators remain essential for quantifying antimicrobial exposure, they provide limited information on the economic importance of antimicrobial classes and animal sectors. Although some jurisdictions publish monetary sales data for veterinary medicinal products, including antimicrobials [18], to the best of our knowledge, such data have rarely been analyzed alongside antimicrobial sales-volume data to support the interpretation of veterinary AMU surveillance. Accordingly, the objective of this study was to assess, using JVARM data from 2011 to 2024, whether the joint analysis of sales volume and sales value could provide a more comprehensive understanding of veterinary antimicrobial sales patterns in Japan than sales volume alone.

## 2. Results

### 2.1. Sales Volume and Sales Value Temporal Patterns

**Figure 1:**
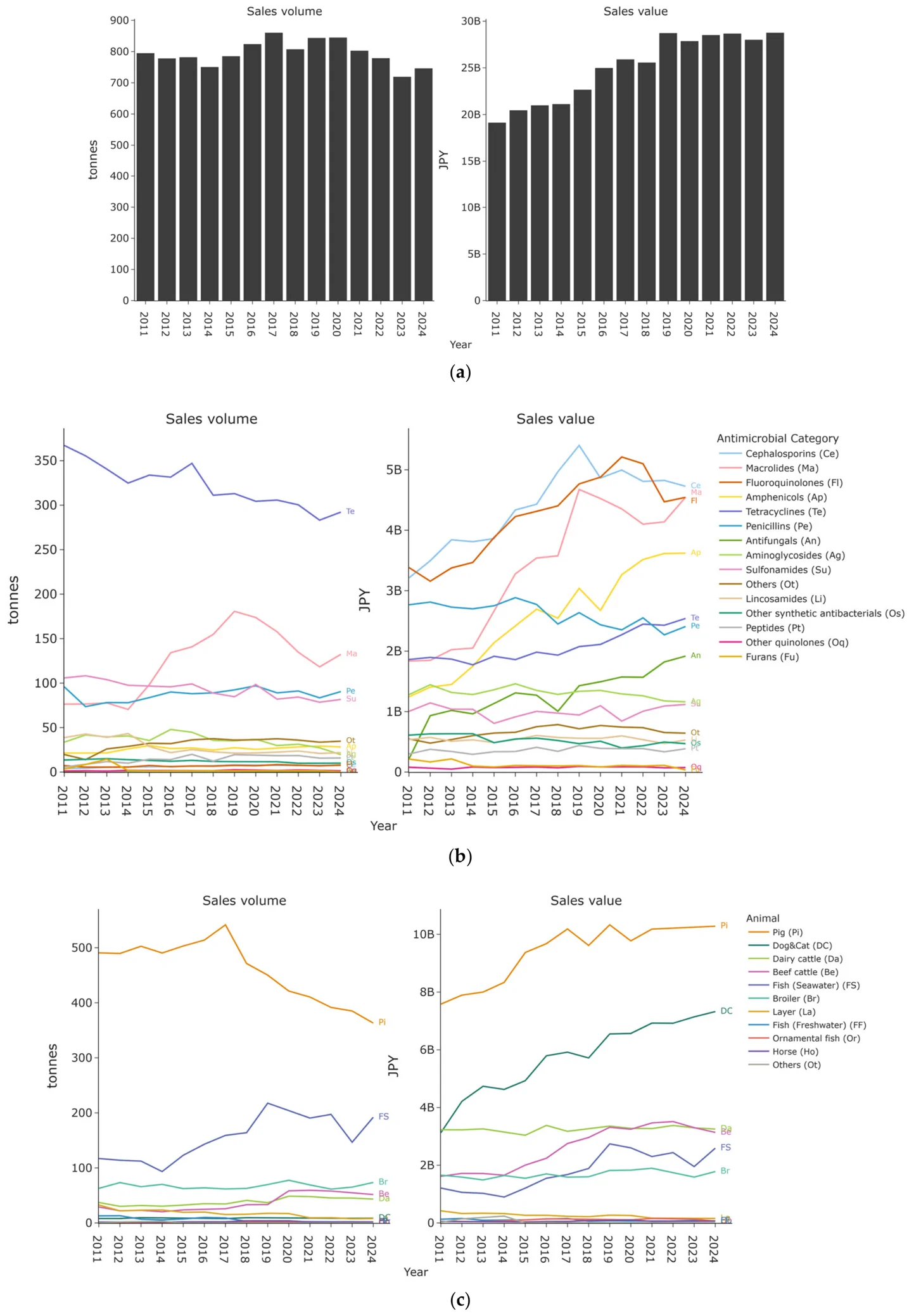
Temporal trends in veterinary antimicrobial sales volume and sales value in Japan, 2011–2024. (**a**) Annual total sales volume and sales value. (**b**) Annual sales volume and sales value by antimicrobial class. (**c**) Annual sales volume and sales value by animal species.

Figure 1a shows annual sales volume and sales value. Total sales volume increased from 794 tonnes in 2011 to a peak of 859 tonnes in 2017. It then declined to 745 tonnes in 2024. In contrast, total sales value increased from JPY 19.1 billion in 2011 to JPY 28.7 billion in 2024. The value in 2024 was the highest during the study period. After 2017, sales volume declined while sales value continued to increase.

Sales volume and sales value also differed across antimicrobial classes (Figure 1b). In terms of sales volume, tetracyclines remained the dominant class throughout the period. However, their sales volume declined from 367 tonnes in 2011 to 292 tonnes in 2024. In 2024, the next largest classes by sales volume were macrolides at 132 tonnes, penicillins at 90 tonnes, and sulfonamides at 82 tonnes. The ranking differed when sales value was considered. In 2024, cephalosporins had the highest sales value at JPY 4.73 billion. Macrolides and fluoroquinolones followed at JPY 4.55 billion and JPY 4.54 billion, respectively. Amphenicols and tetracyclines had sales values of JPY 3.62 billion and JPY 2.54 billion, respectively. Several classes showed different patterns between the two measures. Cephalosporins and fluoroquinolones ranked among the highest classes by sales value despite their relatively low sales volumes. Their sales volumes in 2024 were 7.4 tonnes and 7.9 tonnes, respectively. Tetracycline sales volume declined during the study period, whereas its sales value increased from JPY 1.86 billion in 2011 to JPY 2.54 billion in 2024. Amphenicols also showed a marked difference between the two measures. Their sales volume increased only modestly, from 21.3 tonnes in 2011 to 28.2 tonnes in 2024. In contrast, their sales value increased from JPY 1.25 billion to JPY 3.62 billion over the same period. Macrolides were prominent under both measures. Their sales volume increased from 76.4 tonnes in 2011 to 132.3 tonnes in 2024. Their sales value also increased from JPY 1.84 billion to JPY 4.55 billion over the same period.

Clear differences between the two measures were also observed across animal species (Figure 1c). By sales volume, pigs were the dominant sector in both 2011 and 2024, although pig-associated sales volume declined from 491 tonnes to 363 tonnes over this period. The second-largest volume-based sector was fish raised in seawater, which increased markedly from 117 tonnes in 2011 to 192 tonnes in 2024. In contrast, the leading sectors by sales value in 2024 were pigs (JPY 10.28 billion) and dogs and cats (JPY 7.32 billion). Sales volume for dogs and cats changed little, from 8.1 tonnes in 2011 to 8.6 tonnes in 2024. In contrast, their sales value increased from JPY 3.12 billion to JPY 7.32 billion over the same period. Pig-associated sales volume decreased from 491 tonnes in 2011 to 363 tonnes in 2024. In contrast, pig-associated sales value increased from JPY 7.58 billion to JPY 10.28 billion over the same period. In addition, beef cattle showed an overall parallel increase between 2011 and 2024 in both sales volume (29 to 52 tonnes) and sales value (JPY 1.62 to JPY 3.14 billion). Together, these results show that differences between sales volume and sales value were observed not only across antimicrobial classes but also across animal sectors.

### 2.2. Sales Volume and Sales Value Describe Distinct Market Dimensions

**Figure 2:**
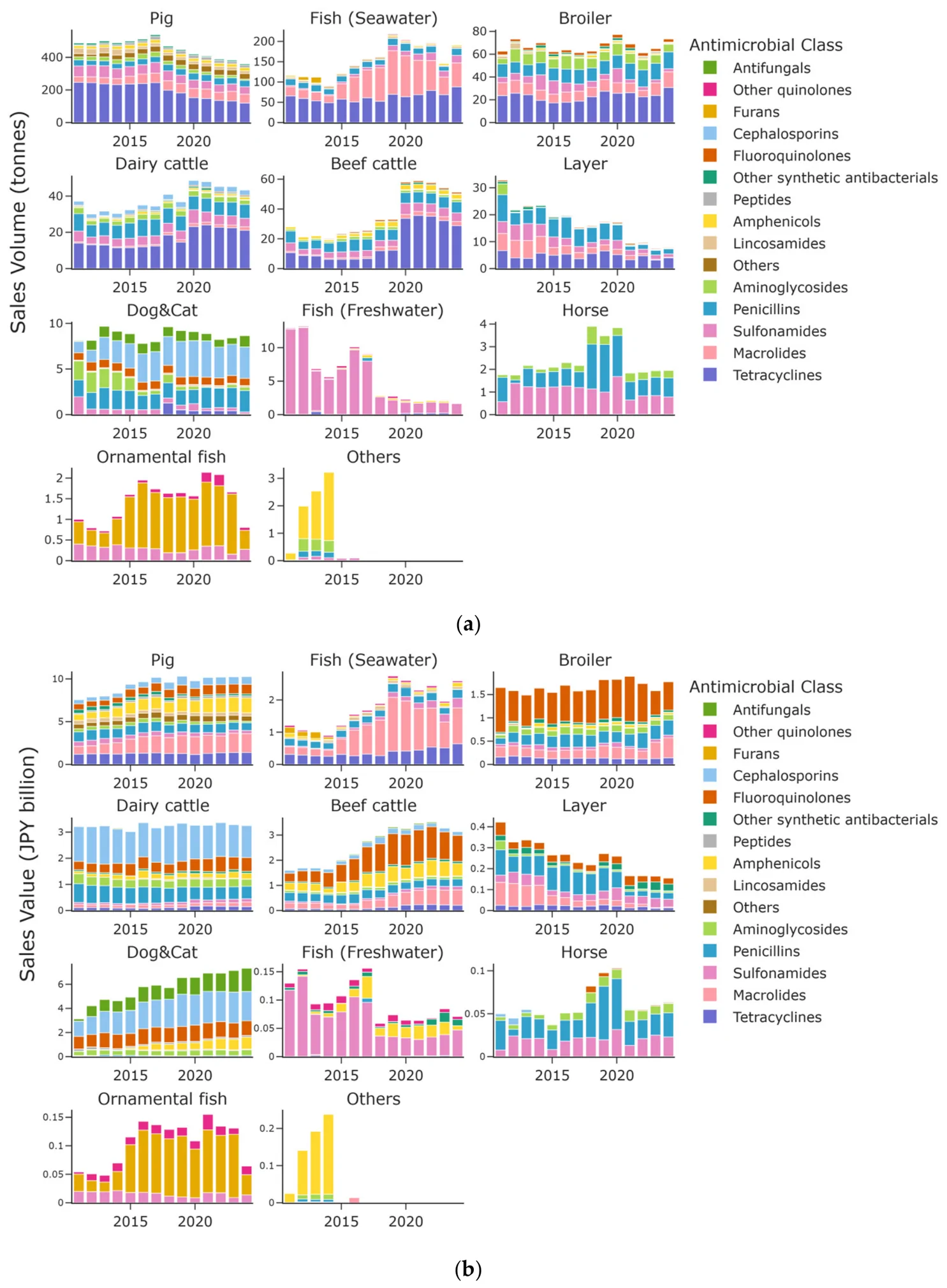
Antimicrobial class composition of veterinary antimicrobial sales volume (**a**) and sales value (**b**) by animal species.

Figure 2 shows annual sales volume and sales value by antimicrobial class within each animal sector.

With respect to sales volume, the dominant class differed across sectors (Figure 2a). In pigs, tetracyclines remained the largest class by sales volume throughout the study period. In 2024, their sales volume was 120 tonnes, followed by macrolides at 55 tonnes, sulfonamides at 46 tonnes, and penicillins at 39 tonnes. In fish raised in seawater, tetracyclines (88 tonnes) and macrolides (59 tonnes) together accounted for most of the sector’s sales volume in 2024. In broilers, tetracyclines were also the largest class by volume (31 tonnes). In dairy cattle and beef cattle, tetracyclines were likewise the largest class by volume in 2024, accounting for 21 tonnes and 29 tonnes, respectively. In dogs and cats, total sales volume was relatively low in 2024 at 8.6 tonnes, with cephalosporins accounting for 3.5 tonnes and penicillins for 2.4 tonnes. Across sectors, tetracyclines were the largest class by volume in several livestock sectors, whereas cephalosporins were the largest class in dogs and cats.

With respect to sales value, the dominant class was not always the same as that for sales volume (Figure 2b). In pigs, macrolides were the largest class by sales value in 2024, at JPY 2.21 billion. In broilers, fluoroquinolones were the largest by sales value, at JPY 0.61 billion. In dairy cattle, cephalosporins were the largest by sales value, at JPY 1.22 billion. In beef cattle, fluoroquinolones were the largest by sales value, at JPY 1.04 billion. In fish raised in seawater, macrolides were the largest by sales value, at JPY 1.11 billion. In dogs and cats, cephalosporins were the largest class under both metrics in 2024, accounting for JPY 2.44 billion in sales value. Taken together, Figure 2 indicates that antimicrobial class composition differed between sales volume and sales value in several animal sectors.

### 2.3. Volume–Value Positioning of Antimicrobial Classes

**Figure 3:**
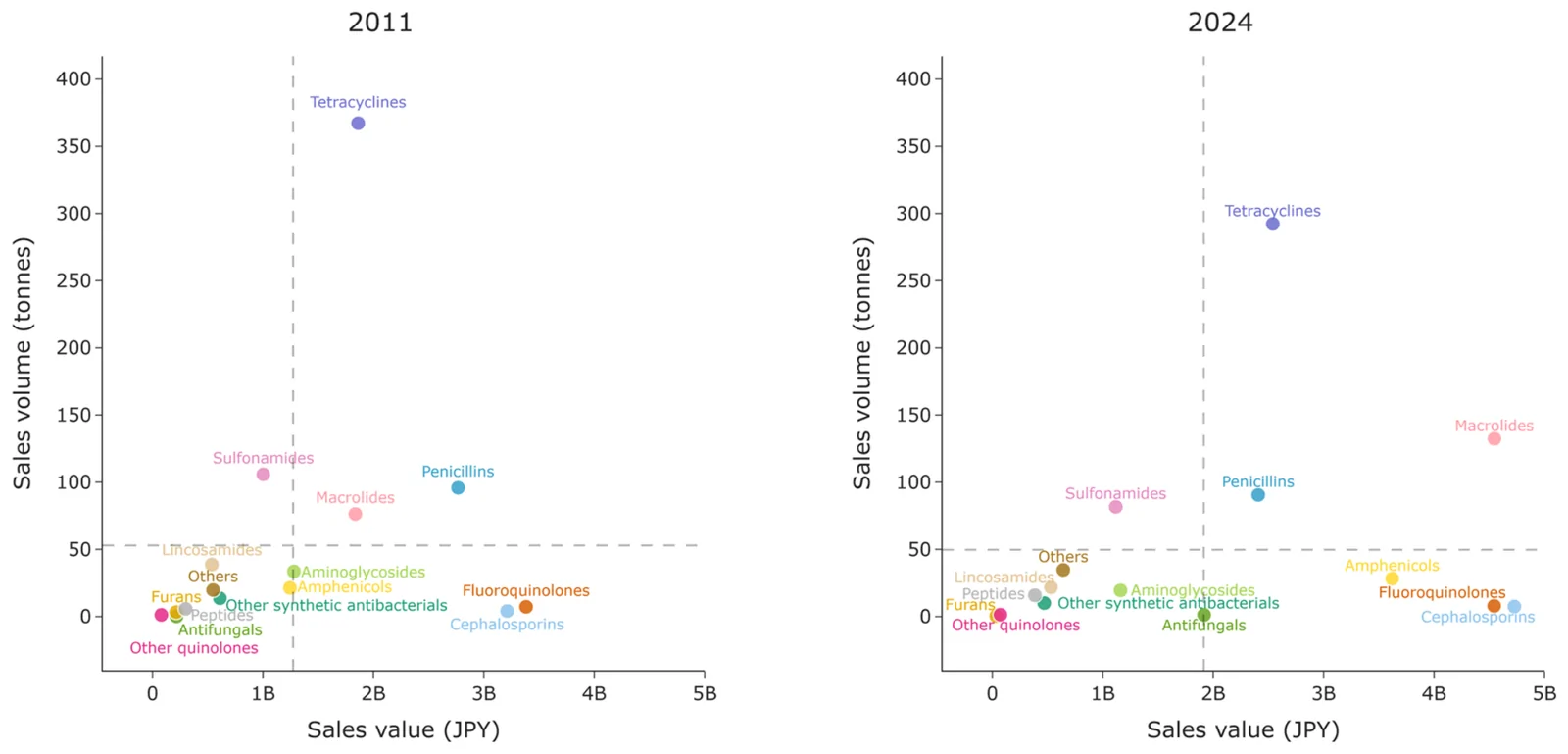
Volume–value positioning of veterinary antimicrobial classes in 2011 and 2024. Dashed reference lines indicate the mean sales value and mean sales volume across antimicrobial classes for each year.

Figure 3 illustrates the relationship between sales volume and sales value by antimicrobial class in 2011 and 2024. Each panel shows antimicrobial classes in a two-dimensional volume–value space. The dashed reference lines indicate the mean sales volume and mean sales value across classes in the corresponding year. The resulting four-quadrant classification distinguishes classes with high or low sales volume relative to the class mean and high or low sales value relative to the class mean.

In 2024, tetracyclines, macrolides, and penicillins were located in the high-volume/high-value quadrant. Sulfonamides were the only class in the high-volume/low-value quadrant. The low-volume/high-value quadrant included amphenicols, antifungals, cephalosporins, and fluoroquinolones. The low-volume/low-value quadrant included aminoglycosides, furans, lincosamides, other quinolones, other synthetic antibacterials, peptides, and others.

The comparison between 2011 and 2024 showed that the positions of several major classes were broadly stable. Tetracyclines, macrolides, and penicillins remained in the high-volume/high-value quadrant in both years. Macrolides moved markedly upward and to the right within this quadrant, with sales value increasing more strongly than sales volume. Sulfonamides remained in the high-volume/low-value quadrant. Cephalosporins and fluoroquinolones remained in the low-volume/high-value quadrant. Amphenicols and antifungals shifted from the low-volume/low-value quadrant in 2011 to the low-volume/high-value quadrant in 2024. Aminoglycosides showed the opposite pattern, moving from the low-volume/high-value quadrant in 2011 to the low-volume/low-value quadrant in 2024.

### 2.4. Concordance and Divergence Between Standardized Sales Volume and Sales Value Trajectories

**Figure 4:**
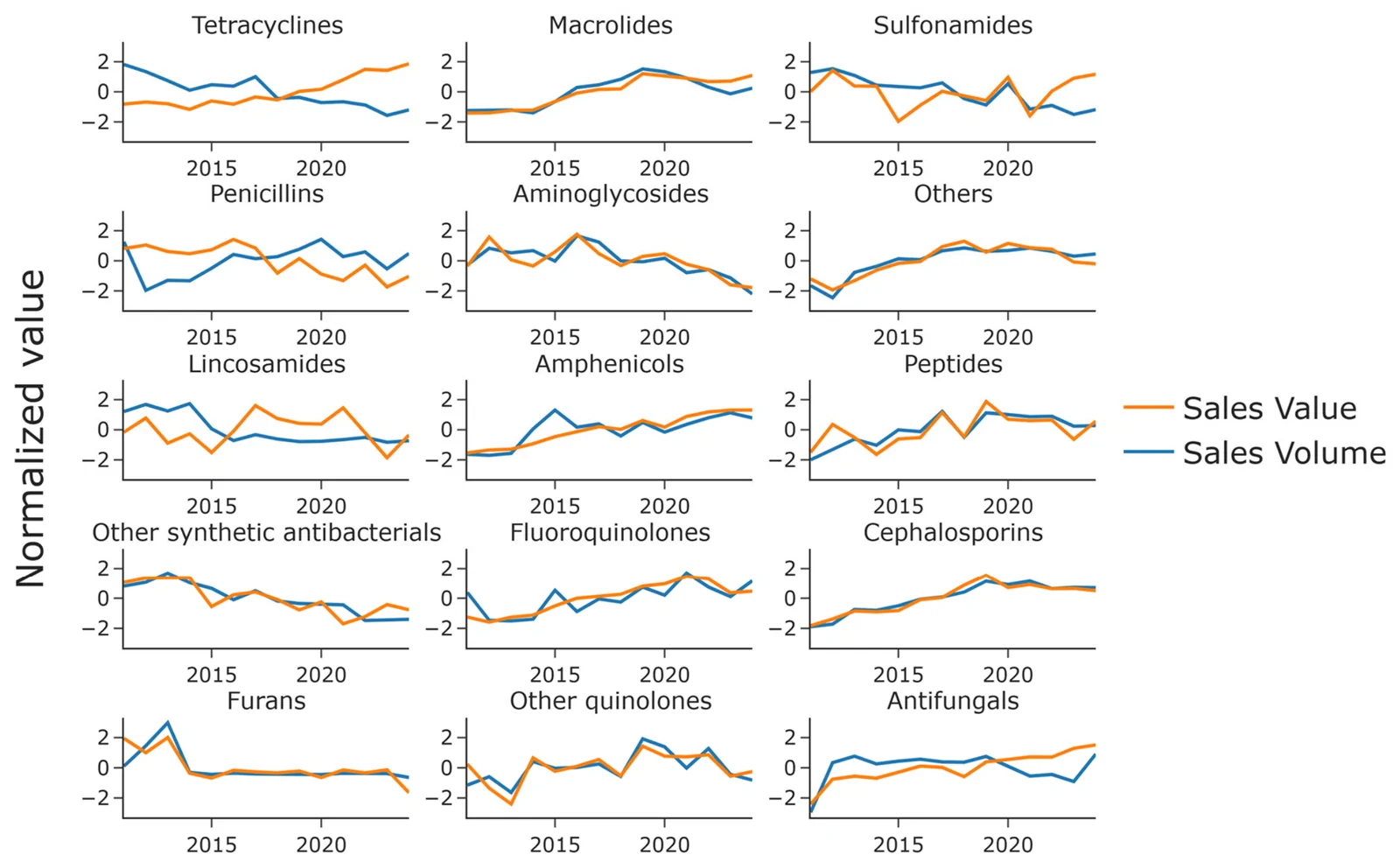
Standardized temporal trajectories of sales volume and sales value by antimicrobial class.

Figure 4 compares standardized annual sales volume and sales value trajectories for each antimicrobial class from 2011 to 2024. By standardizing each measure within each class, the figure allows comparison of whether sales volume and sales value changed in similar directions over time or followed divergent temporal patterns.

Based on visual comparison, the standardized sales-volume and sales-value trajectories were broadly concordant for some antimicrobial classes but diverged for others. Classes such as macrolides, aminoglycosides, others, amphenicols, peptides, other synthetic antibacterials, fluoroquinolones, cephalosporins, furans, and other quinolones showed broadly concordant movement. In contrast, tetracyclines, sulfonamides, penicillins, lincosamides, and antifungals showed differing patterns between the two measures.

Among the classes showing divergent trajectories, tetracyclines showed a decline in sales volume from 367 tonnes in 2011 to 292 tonnes in 2024, whereas sales value increased from JPY 1.86 billion to JPY 2.54 billion. For sulfonamides, the standardized sales-volume and sales-value trajectories were broadly concordant until 2014. They diverged in 2015, when sales value decreased from JPY 1.04 billion to JPY 0.81 billion, while sales volume changed little from 97.6 tonnes to 96.7 tonnes. The trajectories converged again by 2017 but diverged after 2022. Sales value increased from JPY 1.01 billion in 2022 to JPY 1.12 billion in 2024, without a corresponding increase in sales volume. For penicillins, sales volume generally increased after 2012 and reached 97.0 tonnes in 2020. In contrast, sales value peaked at JPY 2.88 billion in 2016 and then declined to JPY 2.41 billion in 2024. For lincosamides, the standardized sales volume trajectory remained above the standardized sales value trajectory from 2011 to 2015. The relationship reversed in 2016. In 2016, sales value increased from JPY 0.49 billion to JPY 0.54 billion, whereas sales volume decreased from 28.7 tonnes to 21.9 tonnes. For antifungals, sales volume remained close to 1 tonne in most years from 2012 onward. It decreased between 2021 and 2023 but increased in 2024. In contrast, sales value increased from JPY 0.22 billion in 2011 to JPY 1.92 billion in 2024.

### 2.5. Class-Specific Elasticity and Marginal Effect Estimates

**Figure 5:**
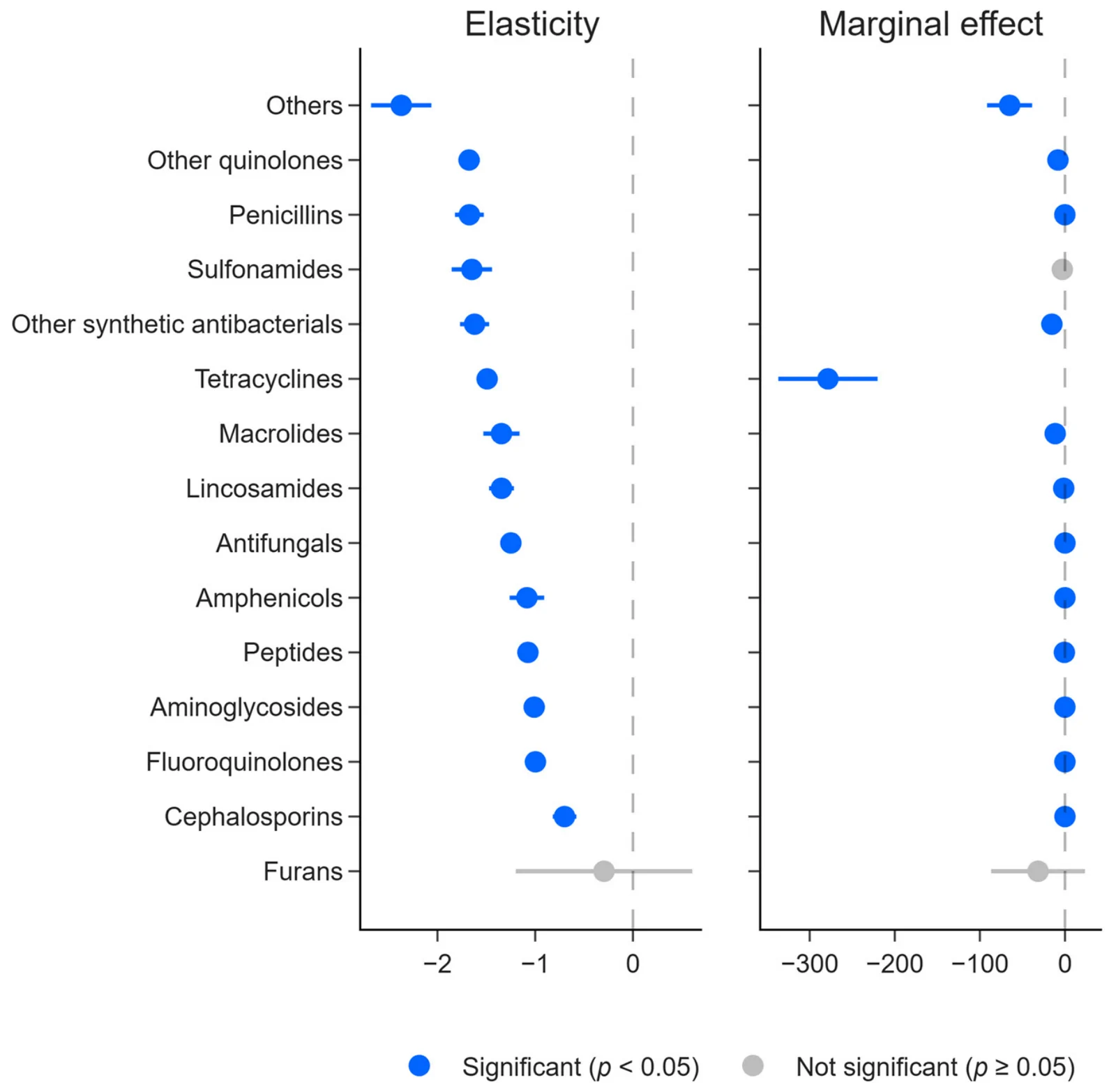
Class-specific estimates of elasticity and marginal effects for sales volume in relation to the implied unit price. Points indicate coefficient estimates, error bars indicate 95% confidence intervals, and the vertical dashed line in each panel indicates zero.

Figure 5 summarizes class-specific price elasticity and marginal effect estimates for the relationship between sales volume and the implied unit price (JPY/g). The implied unit price (JPY/g) was calculated as sales value divided by sales volume. Elasticity was estimated using log–log models with year fixed effects, and marginal effects were estimated using linear models with year fixed effects. To facilitate interpretation, estimates are presented with reference to the 2024 WHO List of Medically Important Antimicrobials (WHO MIA) [19], which categorizes antimicrobials according to their importance for human medicine. The antimicrobial substances marketed in Japan were grouped into the analytical classes shown in Table 1. These classes generally aligned with the WHO MIA categories. However, some analytical classes included substances assigned to different WHO MIA categories and therefore did not correspond exactly to a single WHO MIA category. Antifungals were not covered by the WHO MIA framework.

Elasticity estimates were negative across all classes and were statistically significant for all classes except furans. Among classes containing or related to WHO highest-priority critically important antimicrobial agents, cephalosporins, fluoroquinolones, peptides, and other quinolones had elasticity estimates of −0.70, −1.00, −1.07, and −1.68, respectively. Among classes related to WHO critically important antimicrobials, aminoglycosides and macrolides had elasticity estimates of −1.01 and −1.35, respectively. Among classes related to WHO highly important antimicrobials, amphenicols, lincosamides, tetracyclines, sulfonamides, and penicillins had elasticity estimates of −1.09, −1.34, −1.49, −1.65, and −1.67, respectively. Furans, related to WHO important antimicrobials, had an elasticity estimate of −0.30. Among the remaining antibacterial categories, other synthetic antibacterials and the others category had elasticity estimates of −1.62 and −2.37, respectively. Antifungals had an elasticity estimate of −1.25.

Marginal effect estimates were also negative for all classes. Furans and sulfonamides did not show statistically significant marginal effects, whereas all remaining classes showed statistically significant negative marginal effects. Among classes containing or related to WHO highest-priority critically important antimicrobial agents, cephalosporins, fluoroquinolones, peptides, and other quinolones had marginal effect estimates of −0.03, −0.05, −0.73, and −8.26 kg per 1 JPY/g, respectively. Among classes related to WHO critically important antimicrobials, aminoglycosides and macrolides had marginal effect estimates of −0.09 and −11.49 kg per 1 JPY/g, respectively. Among classes related to WHO highly important antimicrobials, amphenicols, penicillins, lincosamides, sulfonamides, and tetracyclines had marginal effect estimates of −0.08, −0.14, −1.49, −3.11, and −278.55 kg per 1 JPY/g, respectively. Furans, related to WHO important antimicrobials, had a marginal effect estimate of −31.64 kg per 1 JPY/g. Among the remaining antibacterial categories, other synthetic antibacterials and the others category had marginal effect estimates of −15.41 and −65.07 kg per 1 JPY/g, respectively. Antifungals had a marginal effect estimate of −0.0016 kg per 1 JPY/g.

## 3. Discussion

The first major finding is that veterinary antimicrobial sales showed divergent trends by metric: sales volume peaked in 2017 and then declined, whereas sales value continued to increase. This divergence has important implications because the forces shaping antimicrobial sales—such as disease burden, resistance patterns, product availability, and prescribing or purchasing behaviours—may be expressed differently in volume and value. Because weight-based surveillance captures the mass of antimicrobials distributed, it is well suited to assessing ecological selection pressure [20] but may overlook growing commercial reliance on particular classes or sectors. The present framework addresses this gap by combining quantity-based assessment with value-based analysis of economic prominence across antimicrobial classes and sectors. A recent study of human antibiotic sales in 62 countries reported divergent trends between 2013 and 2023, with sales volume increasing and inflation-adjusted expenditure decreasing [21,22]. The authors discussed this divergence as potentially reflecting changes in market composition, including the availability of lower-cost products. They also noted the limited consideration of prices and affordability in previous quantity-based studies and highlighted the need to examine pricing and spending dynamics in food-animal and agricultural markets [21]. The human-sector study thus illustrates how considering both the quantity and monetary value of antibiotic sales can reveal different aspects of antibiotic markets. This analytical perspective is also relevant to veterinary antimicrobial surveillance.

A second major finding is that the antimicrobial classes that dominate by volume are not necessarily the same as those that dominate by sales value. Fluoroquinolones and cephalosporins were notable examples, accounting for relatively small sales volumes but substantial sales values. The two-dimensional analysis characterized each class according to its relative sales volume and sales value, thereby revealing differences among antimicrobial classes that would not be apparent from sales volume alone. This information may be useful when considering stewardship interventions targeting specific antimicrobial classes. Such measures may also have implications for pharmaceutical companies involved in the development and supply of veterinary antimicrobials [23]. Pharmaceutical companies are important stakeholders in antimicrobial stewardship and can contribute to the responsible use of antimicrobials through a range of activities [24]. Understanding the monetary importance of each class could therefore help anticipate the potential stakeholder and supply-side implications of class-specific interventions.

The relative prominence of animal sectors and of antimicrobial classes within each sector also differed between sales volume and sales value. Pigs remained the dominant sector by sales volume, consistent with our previous study showing the prominence of the pig sector in veterinary antimicrobial use in Japan [25]. Nevertheless, our subsequent analysis showed that antimicrobial use in pigs declined from 2017 onward [17]. The present study adds a sales value perspective to this volume-based trend, showing that the decline in pig-associated sales volume was not accompanied by a corresponding decline in sales value. Although fish raised in seawater ranked second by sales volume, its sales value ranked sixth in 2024. Both measures increased from around 2014, relating to antimicrobial use associated with the treatment of type II α-haemolytic lactococcosis, which emerged around 2013 and against which the conventional vaccine provided limited protection at that time [15]. Companion animals showed a clearer contrast, accounting for only a small proportion of total sales volume but ranking second among animal sectors by sales value in 2024. This market prominence may partly reflect the distinct context of companion-animal medicine. In this sector, individualized and increasingly advanced diagnostic and treatment options are available, while owner attachment may influence expectations for access to such care [26]. Within individual animal sectors, the classes that dominated by volume also frequently differed from those that dominated by value. Tetracyclines were the largest class by sales volume in several sectors, consistent with our previous quantity-based analysis of veterinary antimicrobial use in Japan [17]. However, the addition of sales value revealed different value-based leading classes in all the sectors. These differences may partly reflect variation among animal sectors in disease profiles, treatment practices, approved indications, and the types of products used. A European livestock study documented related variation among animal sectors in group-treatment practices and routes of administration [27].

The divergence between standardized sales volume and sales value trajectories also carried important interpretive meaning. Under stable unit-price and product-mix conditions, the two trajectories would be expected to move broadly in parallel. When they diverge, the divergence may indicate changes in the underlying market structure. Tetracyclines provided the clearest example of this pattern: their sales volume declined, whereas their sales value increased. This divergence highlights the additional insight provided by sales value, suggesting that the economic importance of tetracyclines remained high and may even have increased during the study period. For this reason, the tetracycline divergence prompted a more detailed within-class decomposition. The within-class decomposition suggested that the tetracycline divergence reflected two temporally distinct processes: an early shift in active-ingredient mass toward doxycycline and a later increase in doxycycline’s implied unit value (Supplementary Figure S1). By sales volume, doxycycline’s share increased from 2011 to 2017 but changed little thereafter. By sales value, however, its share rose more substantially. This later increase appears to be consistent with an increase in doxycycline’s implied unit value, calculated as sales value divided by sales volume, which rose from 10.1 JPY/g in 2017 to 17.8 JPY/g in 2024, rather than with a further expansion in its volume share. Regarding the early shift, doxycycline had a substantially higher implied unit value than the other major tetracyclines in 2017: 10.1 JPY/g for doxycycline, compared with 4.1 JPY/g for oxytetracycline and 4.1 JPY/g for chlortetracycline. A notable observation was that this ordering of implied unit value was broadly consistent with differences in DDDvet, particularly in pigs, the largest tetracycline-consuming sector. Doxycycline has lower DDDvet values than oxytetracycline and chlortetracycline across major food-producing species. For oral use, the DDDvet values for doxycycline, oxytetracycline, and chlortetracycline are 11, 26, and 31 mg/kg in pigs; 10, 20, and 22 mg/kg in cattle; and 15, 39, and 30 mg/kg in broilers, respectively [28]. Because doxycycline has lower DDDvet values, the same one-gram amount represents more standardized daily treatment units than oxytetracycline or chlortetracycline. This difference in dose intensity may partly contribute to, or at least be consistent with, the higher implied unit value observed for doxycycline. Thus, in this example, the divergence between volume and value functioned as a practical screening signal that led to the identification of a substance-level shift and increased implied unit values of specific substances that would have been less apparent from aggregate sales volume alone. To provide clinical context, tetracyclines, including doxycycline, are frequently used to treat respiratory diseases in pigs [29], although several other antimicrobial classes are also available for the same indications [30]. Divergent sales volume trends among antimicrobials used for similar indications may generate hypotheses about potential substitution. Such hypotheses cannot be evaluated using the aggregated sales data used in this study and would require prescription-level information.

The elasticity and marginal effect analysis provides an additional perspective on market responsiveness. These analyses are used to characterize how sales volume varies with changes in price [31,32]. In standard economic interpretation, an elasticity estimate closer to zero indicates weaker responsiveness to price changes, potentially reflecting greater necessity or limited substitutability [32]. By contrast, a more strongly negative elasticity indicates that demand decreases more clearly as price increases, suggesting greater substitutability or scope for adjustment. Elasticity expresses proportional responsiveness and, because it is unit-free, allows comparisons across products with different market sizes. In our analysis, we observed elasticities closer to zero for antimicrobial classes generally considered to be of greater clinical importance and more strongly negative elasticities for classes with broader therapeutic applicability. The estimated elasticities were relatively low for cephalosporins and fluoroquinolones, whereas stronger negative estimates were obtained for sulfonamides and penicillins. This pattern is broadly consistent with the WHO MIA framework, in which cephalosporins and fluoroquinolones contain or are related to antimicrobial classes categorized as highest-priority critically important antimicrobials, whereas sulfonamides and penicillins are categorized as highly important antimicrobials [19]. To further examine this interpretation, cephalosporins were disaggregated by generation. In the main analysis, cephalosporins were treated as a single analytical class, although the class included both first- and second-generation cephalosporins and third- and higher-generation cephalosporins. Under the WHO MIA framework, these subclasses differ in importance: third- and higher-generation cephalosporins are classified as highest-priority critically important antimicrobials, whereas first- and second-generation cephalosporins are classified as highly important antimicrobials. When separated accordingly, third- and higher-generation cephalosporins showed a much weaker elasticity estimate than first- and second-generation cephalosporins, with estimates of −0.25 (95% CI: −0.35 to −0.16) and −1.27 (95% CI: −1.44 to −1.10), respectively. The WOAH List of Antimicrobial Agents of Veterinary Importance has a similar role to the WHO MIA framework but was developed from a veterinary-medicine perspective to provide information on antimicrobial agents and classes authorized for use in animals and their importance for treating common infectious diseases in animals [33]. According to the WOAH list, the classification of fluoroquinolones and cephalosporins was broadly similar to that in the WHO MIA framework: fluoroquinolones and third- and higher-generation cephalosporins are classified as veterinary critically important antimicrobial agents, whereas first- and second-generation cephalosporins are classified as veterinary highly important antimicrobial agents. In contrast to the WHO MIA framework, the WOAH framework classifies penicillins and sulfonamides as veterinary critically important antimicrobial agents. Thus, although WHO and WOAH categories are based on clinical and stewardship considerations rather than market behaviour, some overlap was observed between classes with lower estimated elasticities and classes prioritized in the WHO and WOAH frameworks. This suggests that elasticity may provide a quantitative market-based signal related to limited substitutability in veterinary antimicrobial use. These findings can also be considered alongside antimicrobial sales trends and stewardship measures in Japan. Following the introduction of the first National Action Plan on Antimicrobial Resistance in 2016 [34], tetracycline sales declined overall, particularly in pigs, with sales for pigs in 2022 falling to half the 2001 level [15]. The second National Action Plan set a target of no more than 27 tonnes in 2027 for designated second-line antimicrobials used in livestock, including fluoroquinolones and third-generation cephalosporins [14]. Their sales volume remained relatively stable from 2020 to 2022 and was within this target in 2022 [15]. These observations broadly correspond to the lower elasticity estimated for fluoroquinolones and cephalosporins and the more strongly negative elasticity estimated for tetracyclines. Trends in European sales volume provide a useful comparison. The ESVAC report showed substantial reductions between 2011 and 2022 in major high-volume classes, with tetracyclines, penicillins, and sulfonamides declining by 70.7%, 30.2%, and 61.5%, respectively. In addition, sales volumes of fluoroquinolones and third- and fourth-generation cephalosporins also declined by 25% and 49%, respectively [5]. These reductions may partly reflect sustained country-specific policies and stewardship initiatives that specifically targeted these critically important classes [35,36]. This comparison highlights the importance of regulatory and stewardship context when interpreting elasticity estimates.

Unlike elasticity, which expresses responsiveness as a percentage change, marginal effects express the estimated difference in sales volume in kilograms. In this study, they indicate the estimated difference in sales volume, in kilograms, associated with a JPY 1/g higher implied unit price. Marginal effects may therefore be more directly relevant than elasticity when discussing market-based instruments (MBIs) in veterinary antimicrobial stewardship. MBIs, particularly taxation, have been proposed as tools to reduce antimicrobial use in food-producing animals, but only a small number of countries have implemented such policies in practice [37]. Belgium and Denmark both introduced differentiated tax measures on veterinary antimicrobials, with charges linked to the active antimicrobial compound and higher rates for selected critically important classes such as fluoroquinolones and cephalosporins (and, in Belgium, also macrolides) [38,39]. The Danish Veterinary and Food Administration (DVFA) does not consider the taxes themselves to change the usage pattern [39], whereas a review by the Belgian Health Care Knowledge Centre (KCE) found that the impact of this measure on antibiotic use is unclear and that the taxes are presumably too low to influence use or stimulate alternative treatments and/or prevention measures [40]. In our data, cephalosporins and fluoroquinolones showed small estimated marginal effects in absolute terms, suggesting that increases in implied unit price would translate into only limited reductions in sales volume for these classes. It remains unclear whether these findings from Japan are applicable to other countries with different veterinary antimicrobial markets and policy settings. However, this pattern is broadly consistent with observations from Belgium and Denmark and may help generate hypotheses about the limited effects of price-based interventions targeting critically important antimicrobial classes.

This study also has limitations. First, sales data are not the same as use data; sales data do not directly measure the quantities of antimicrobials actually administered to animals. Second, sales value may reflect not only unit price in a narrow sense but also bulk pricing, formulation mix, route of administration, species-specific product portfolios, and other commercial factors. Third, the elasticity analysis was based on implied unit-price information reconstructed from aggregated value and volume and should therefore be interpreted as descriptive rather than causal. Fourth, the analyses presented in Section 2.3, Section 2.4 and Section 2.5 focused on overall volume–value patterns and responsiveness in veterinary antimicrobial sales in Japan and were not stratified by animal category. This study was designed to first characterize overall national patterns before conducting more detailed sector-specific analyses. Fifth, the present analyses were conducted using aggregated sales data and did not consider differences in disease profiles, treatment indications, or prescribing practices across animal species. Sixth, the findings reflect the structure of Japan’s animal health sectors, veterinary regulatory framework, and antimicrobial market and supply system and may not be directly generalizable to other countries.

Despite these limitations, this study provides a useful complementary extension to routine veterinary AMU surveillance. Adding sales value metrics to existing mass-based monitoring can provide market context for interpreting surveillance signals. The analytical approach could be applied in other countries. Future studies could extend the analyses presented in Section 2.3, Section 2.4 and Section 2.5 by stratifying the data into livestock, aquaculture, and companion animal sectors to examine sector-specific volume–value patterns and responsiveness. Such studies could also examine the relationships between these market-contextual indicators and resistance trends and further refine the sales value metrics by developing biomass-adjusted measures, such as JPY/biomass.

## 4. Materials and Methods

### 4.1. Study Design and Data Source

This retrospective observational study analyzed routinely collected annual veterinary antimicrobial sales data from JVARM over the 14-year period from 2011 to 2024. The data were obtained from the publicly available Annual Reports of Sales Amount and Sales Volume of Antibiotics, Synthetic Antibacterials, Anthelmintics, and Antiprotozoals published by the National Veterinary Assay Laboratory as part of JVARM [41]. Sales data are reported annually by marketing authorization holders in accordance with the Pharmaceutical and Medical Device Act. The reports provide sales data aggregated by active substance rather than data for individual veterinary medicinal products. The reported sales volume and sales value combine data for individual veterinary medicinal products containing the same active substance. Each reported sales entry includes the active substance name, route of administration, sales volume expressed in kilograms of active ingredient, sales value expressed in Japanese yen (JPY), and estimated allocation proportions by animal category.

Data were included if they represented sales of veterinary antimicrobials reported between 2011 and 2024. Anthelmintics and antiprotozoals contained in the source reports were excluded because they were outside the scope of this study. The included antimicrobials were subsequently grouped into the classes shown in Table 1 for analysis.

The source reports provided the estimated proportion of sales allocated to each animal category. Because these proportions occasionally did not sum exactly to one due to rounding, we divided each proportion by the sum of the proportions across all animal categories. We then used the rescaled proportions to allocate sales volume and sales value across animal categories. Animal categories included pigs, broilers, layers, dairy cattle, beef cattle, horses, dogs and cats, fish raised in seawater, fish raised in freshwater, ornamental fish, and other animals.

### 4.2. Descriptive Analyses of Sales Volume, Sales Value, and Volume–Value Positioning

Using the JVARM sales data described in Section 4.1 [41], annual sales volume and sales value were summarized from 2011 to 2024 overall, by antimicrobial class, by animal category, and by antimicrobial class within each animal category. Sales volume was expressed in tonnes of active ingredient, and sales value in billions of JPY. These summaries were used to describe temporal trends in total, by antimicrobial class, and by animal category (Figure 1) and compare antimicrobial class composition within animal categories between sales volume and sales value (Figure 2). As a descriptive analysis, antimicrobial classes were positioned in a two-dimensional volume–value space using class-specific total sales volume and total sales value in 2011 and 2024 (Figure 3). In each of these two years, mean sales volume and mean sales value across classes were used as reference lines to classify classes into high-volume/high-value, high-volume/low-value, low-volume/high-value, and low-volume/low-value groups.

### 4.3. Standardized Temporal Trajectory Analysis

To compare temporal changes in sales volume and sales value within each antimicrobial class, annual class-specific totals were calculated irrespective of animal category. For each antimicrobial class and metric, standardized values (z) were calculated as z = (x − μ)/σ, where x is the annual value and μ and σ are the mean and standard deviation, respectively, of the corresponding metric calculated across the study period. The standardized sales-volume and sales-value trajectories were plotted together for each antimicrobial class (Figure 4), allowing visual comparison of whether the two metrics moved concordantly or diverged over time.

### 4.4. Elasticity and Marginal Effect Analysis

For the elasticity and marginal effect analyses, sales entries from 2011 to 2024 were used, retaining information on year, antimicrobial class, antimicrobial active substance name, route of administration, sales volume, and sales value. Observations with non-positive sales volume or non-positive sales value were excluded.

An implied unit price was calculated for each sales entry as sales value divided by sales volume and expressed as JPY per gram of active ingredient (JPY/g).


$$Implied\;unit\;price\;(JPY/g)=\;\frac{Sales\;value\;(JPY)}{Sales\;volume\;(g)}$$

For each antimicrobial class, elasticity was estimated using a log–log ordinary least-squares model with year fixed effects, as follows. The elasticity was interpreted as the percentage difference in sales volume associated with a 1% higher implied unit price.


$$\log\left(Sales\;volume\;in\;kg\right)=\;Intercept\;+\;Elasticity\;\times \;\log\left(\;Implied\;unit\;price\;\right)+\;\;Year\;fixed\;effects\;+\;Error\;term$$

Marginal effects were estimated using a linear ordinary least-squares model with year fixed effects, as follows. The marginal effect was interpreted as the absolute difference in sales volume, in kilograms, associated with a one JPY/g increase in the implied unit price.


Sales volume in kg = Intercept + Marginal effect × Implied unit price + Year fixed effects + Error term

Both models were fitted separately for each antimicrobial class. Coefficients were estimated by ordinary least squares, with HC1 heteroskedasticity-robust standard errors used to calculate 95% confidence intervals and *p*-values. Statistical significance was assessed at *p* < 0.05. These analyses were intended as descriptive indicators of relative responsiveness rather than as causal estimates of the effect of price on antimicrobial sales.

Class-specific elasticity and marginal-effect estimates were presented with reference to the WHO classification of medically important antimicrobials [19]. The analytical antimicrobial classes used in this study did not always correspond exactly to the WHO categories.

### 4.5. Software

Data wrangling, statistical modeling, and visualization were conducted in Python (v3.11.5) using pandas (v2.2.3) [42], numpy (v2.3.5) [43], statsmodels (v0.14.6) [44], and plotly (v5.18.0) [45].

## 5. Conclusions

Integrated monitoring of veterinary antimicrobial sales volume and sales value provides a richer surveillance picture than sales volume alone. Sales volume alone describes the distribution of antimicrobial mass. Adding sales value provides complementary information on the relative market prominence of antimicrobial classes and animal sectors and how this prominence changes over time. Because stewardship depends not only on how much antimicrobial is used but also on where reliance is concentrated, adding value-based summaries can improve the interpretation of surveillance signals and support more nuanced prioritization. The proposed framework is complementary to existing surveillance systems and could be implemented in jurisdictions where both sales volume and sales value data are available. However, the findings should be interpreted cautiously because sales data do not directly measure the quantities of antimicrobials actually administered to animals. Future studies should further refine sales-value metrics by developing biomass-adjusted measures, such as JPY/biomass.

## Figures and Tables

**Table 1:** Classification of antimicrobials used in this study.

Antimicrobial Class	Antimicrobials
Aminoglycosides	Apramycin, Dihydrostreptomycin, Fradiomycin, Gentamicin, Kanamycin, Spectinomycin, Streptomycin
Amphenicols	Chloramphenicol, Florfenicol, Thiamphenicol
Antifungals	Clotrimazole, Itraconazole, Ketoconazole, Miconazole, Nanafrocin, Nystatin, Pimaricin, Terbinafine
Cephalosporins	Cefalexin, Cefalonium, Cefapirin, Cefazolin, Cefovecin, Cefpodoxime, Cefquinome, Ceftiofur, Cefuroxime
Fluoroquinolones	Danofloxacin, Difloxacin, Enrofloxacin, Lomefloxacin, Marbofloxacin, Norfloxacin, Ofloxacin, Orbifloxacin, Pradofloxacin
Furans	Nifurstyrenic acid, Nitrofurazone
Lincosamides	Clindamycin, Lincomycin, Pirlimycin
Macrolides	Erythromycin, Gamithromycin, Josamycin, Mirosamycin, Spiramycin, Tildipirosin, Tilmicosin, Tulathromycin, Tylosin, Tylvalosin
Other quinolones	Miloxacin, Oxolinic acid
Other synthetic antibacterials	Ormetoprim, Trimethoprim
Others	Bicozamycin, Fosfomycin, Tiamulin, Valnemulin
Penicillins	Amoxicillin, Ampicillin, Aspoxicillin, Benzylpenicillin, Cloxacillin, Dicloxacillin, Mecillinam, Nafcillin, Tobicillin
Peptides	Bacitracin, Colistin, Thiostrepton
Sulfonamides	Homosulfamine, Sulfachlorpyridazine, Sulfadiazine, Sulfadimethoxine, Sulfadimidine, Sulfadoxine, Sulfamerazine, Sulfamethoxazole, Sulfamethoxypyridazine, Sulfamonomethoxine, Sulfamoyldapsone, Sulfaquinoxaline, Sulfisozole
Tetracyclines	Chlortetracycline, Doxycycline, Oxytetracycline

## Data Availability

The data supporting the findings of this study are publicly available from the sources described in the Materials and Methods section and listed in the References.

## Supplementary Materials

The following supporting information can be downloaded at: https://www.mdpi.com/article/10.3390/antibiotics15090908/s1: Figure S1: Substance-level decomposition of tetracycline sales volume, value, and implied unit value.
