# Supplementary material for: Incorporating Sales Value into Weight-Based Veterinary Antimicrobial Use Surveillance: An Analysis of Veterinary Antimicrobial Sales Data in Japan, 2011–2024
Source: Antibiotics (Basel). 2026 Sep 15;15(9):908. doi: 10.3390/antibiotics15090908 (PMC13603399; doi:10.3390/antibiotics15090908)
Supplement: Supplementary file 1 [file antibiotics-15-00908-s001.zip › antibiotics-4511573-supplementary.pdf]

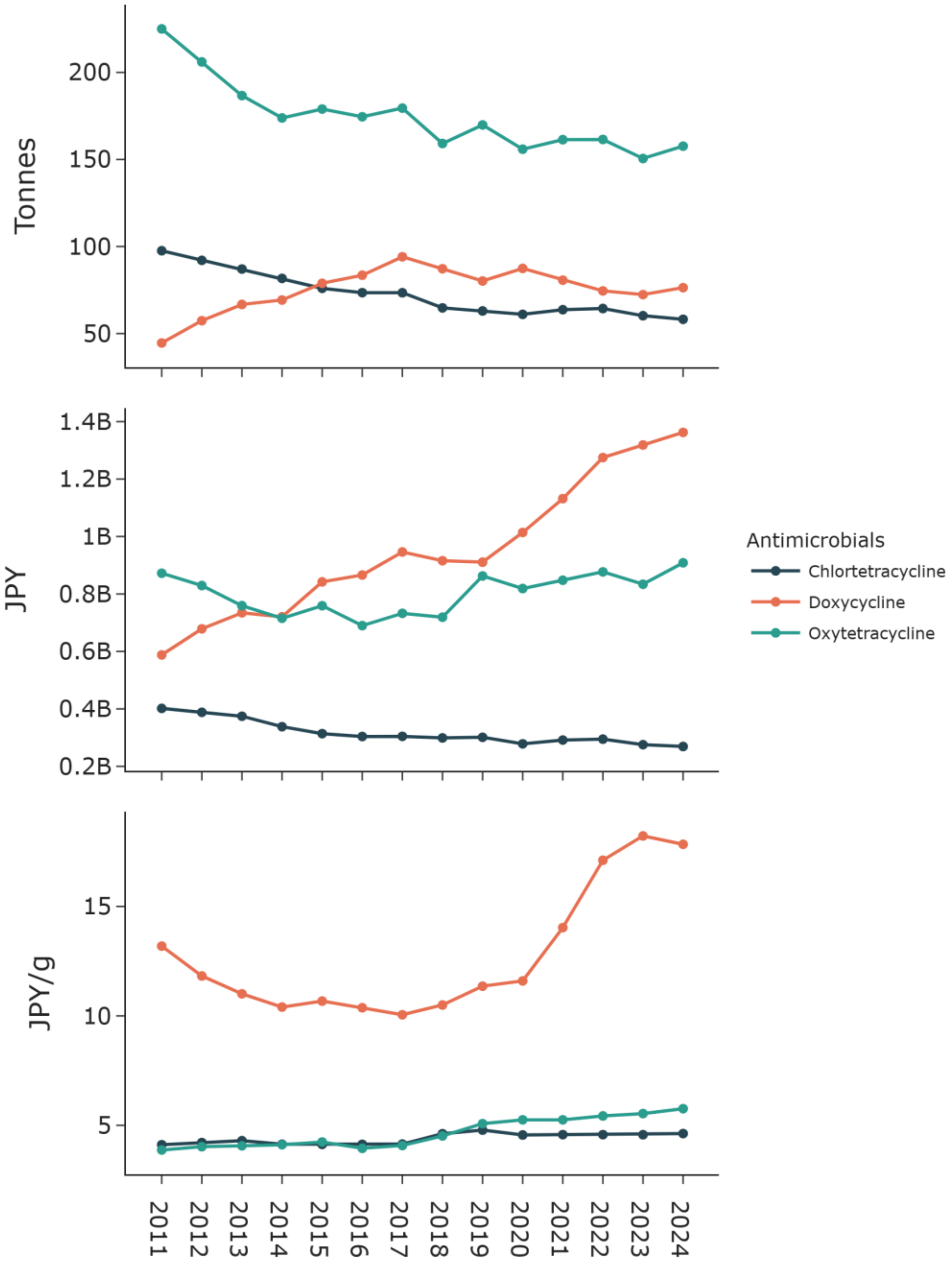

Supplement Figure S1. Substance-level decomposition of tetracycline sales volume, value, and implied unit value.
